# A Multi-centered Retrospective Study on the Efficacy of Pulsed Radiofrequency Nerve Ablation in the Treatment of Recalcitrant Plantar Fasciitis: A Mid-term Outcome

**DOI:** 10.7759/cureus.58021

**Published:** 2024-04-11

**Authors:** Kumarendran Kanesen, Mohd Shahril Jaafar, Azammuddin Alias, Ming Long Kam, Mohd Yusoff B Yahaya

**Affiliations:** 1 Department of Orthopedics and Traumatology, Hospital Sultan Abdul Aziz Shah/University Putra Malaysia, Serdang, MYS; 2 Department of Orthopedics, Hospital Sultan Idris Shah Serdang, Kajang, MYS; 3 Department of Orthopedics and Traumatology, Universiti Teknologi MARA, Shah Alam, MYS

**Keywords:** american orthopedic foot and ankle society (aofas) ankle/hindfoot score, visual analogue scale (vas), electronic medical record (emr), hospital information system (his), recalcitrant plantar fasciitis, pulsed radiofrequency nerve ablation, plantar fasciitis

## Abstract

Background

Plantar fasciitis, a condition marked by persistent and often excruciating heel pain, frequently poses a formidable hurdle when conservative treatment approaches fall short. This multi-centered retrospective study embarks on a journey to explore the potential effectiveness of pulsed radiofrequency nerve ablation (RFNA), an innovative and less invasive procedure, as a novel avenue for treating recalcitrant plantar fasciitis. This investigation centers around a group of 24 patients who have faced the persistence of this challenging ailment. By meticulously scrutinizing patient outcomes and conducting a comprehensive analysis of safety aspects, this study aspires to offer enlightening revelations regarding the promise and practicality of pulsed RFNA as a therapeutic solution for tackling this intricate and tenacious condition.

Methods

This retrospective study included 24 patients who had undergone pulsed RFNA for recalcitrant plantar fasciitis between June 1, 2020, and June 1, 2022, at Hospital Pengajar Universiti Putra Malaysia (HPUPM), Hospital Universiti Teknologi Mara (UiTM), and Hospital Serdang. Patients were selected from the Orthopedic Clinics at HPUPM, Hospital UiTM, and Hospital Serdang and were screened according to the inclusion and exclusion criteria. Patient data was extracted from the hospital information system and electronic medical records. Pre-procedure and post-procedure assessments were conducted at one, three, and six months on the selected patients using the visual analog scale and American Orthopaedic Foot and Ankle Society Ankle-Hindfoot Scoring systems. All selected patient data was traced and tabulated accordingly.

Results

This study evaluates the effectiveness of pulsed RFNA in treating recalcitrant plantar fasciitis in 24 participants (39 feet). Results show a significant reduction in pain and improvement in functionality at one, three, and six months post-RFNA. Demographic factors (age, gender, and specific diagnosis) did not significantly impact outcomes. The study supports pulsed RFNA as an effective treatment for recalcitrant plantar fasciitis, emphasizing consistent benefits across various patient characteristics.

Conclusion

In conclusion, the study demonstrates the notable effectiveness of pulsed RFNA in improving pain reduction and functional outcomes for individuals with recalcitrant plantar fasciitis. The findings, consistent across various demographic factors, support pulsed RFNA as a promising and uniform treatment option for those who do not respond to conservative measures.

## Introduction

Plantar fasciitis is characterized by inflammation of the plantar fascia, leading to severe and debilitating pain [1]. Approximately 20% to 30% of patients experience bilateral involvement of plantar fasciitis [1]. Despite the elusive nature of its exact etiology, pathophysiology shares similarities with Achilles tendinopathy, involving microscopic degenerative injuries, disruptions in the collagen matrix, and microtears, rather than a deficient healing response. This pathogenic process entails localized inflammation and degeneration of the proximal plantar aponeurosis, particularly near its origin at the anteromedial tubercle of the calcaneus [2].

Plantar fasciitis is a prevalent cause of heel pain, particularly impacting middle-aged to elderly individuals and significantly affecting their overall quality of life. While the precise cause of plantar fasciitis often remains elusive, various well-defined intrinsic and extrinsic risk factors contribute to its development. Intrinsic factors encompass age (typically middle-aged), obesity, tightness in the Achilles tendon, pes planus, and pes cavus, while extrinsic factors involve prolonged weight-bearing, running, walking on hard surfaces, and inappropriate footwear choices [3]. The traditional belief attributes the pain associated with plantar fasciitis to inflammation in the region where the plantar fascia attaches near the anteromedial tubercle of the calcaneus. However, pain may stem from diverse sources, including fractures of bony heel spurs, chronic tears within the plantar fascia, and nerve entrapments [3]. Additionally, it can be associated with the chronic strain of the proximal aspect of the plantar fascia, often occurring concurrently with deformities such as pes planus or pes cavus.

In the physical examination, individuals with plantar fasciitis typically exhibit localized tenderness at the anteromedial aspect of the calcaneus. Palpation reveals overall tenderness across the entire plantar surface, extending more into the arch, distinguishing it from heel pain syndrome. In heel pain syndrome, the maximum tenderness is noted just anterior to the calcaneal tuberosity or at the plantar medial heel. The pain can intensify with passive dorsiflexion of the toes or when the patient stands on the tips of their toes [1].

The management of plantar fasciitis encompasses a wide range of approaches, reflecting the diverse proposed causes of the condition. Conservative options, including stretching, physical therapy, anti-inflammatory drugs, steroid injections, arch support, night splints, massage, and modifications to footwear, have demonstrated frequent success [3]. Additionally, alternative modalities like extracorporeal shockwave therapy and cryotherapy have been employed in the treatment of plantar fasciitis. For more severe cases, surgical interventions such as percutaneous, endoscopic, and open partial plantar fascia releases, as well as calcaneal spur resection, have been considered [3]. It is important to note that opinions on the efficacy of these treatments vary, emphasizing that no single approach guarantees relief in every instance.

Recently, radiofrequency nerve ablation (RFNA) treatment has been used for chronic heel pain associated with plantar fasciitis with a success rate of more than 90%. Literature on the use of RFNA on chronic heel pain is very limited [3]. This technique has been used successfully for the treatment of a variety of conditions involving cranial and spinal nerve injuries.

Plantar fasciitis is defined as chronic or recalcitrant, or both when 6 to 12 months of conservative treatment yielded little or no improvement. It was estimated that 10% of patients with acute plantar fasciitis progressed to chronic symptoms [2]. Two types of radio frequencies were used in the treatment of recalcitrant plantar fasciitis: thermal radiofrequency (TRF) and pulsed radiofrequency (PRF). In this study, PRF was chosen as the treatment method due to its minimally invasive nature and non-neurodestructive approach. TRF has been proposed as a method to treat chronic heel pain. The heat partially damaged the nerve and halted pain transmission. The proposed mechanism of action was the desensitization of nerve endings.

PRF, a more recent non-neurodestructive technique, provided pain relief. The relatively long pauses between pulses allowed for heat dissipation, primarily through conduction and convection. This resulted in surrounding tissue reaching a temperature insufficient for neural coagulation. PRF nerve lesioning has proven to be a safe intervention [1,4]. Compared to TRF, the risks of neuritis, deafferentation pain, and neuroma formation were minimal [1]. In this present study, we examined the use of pulsed RFNA for the treatment of recalcitrant plantar fasciitis.

## Materials and methods

This is a retrospective study that included all patients who had undergone pulsed RFNA for recalcitrant plantar fasciitis between June 1, 2020, and June 1, 2022, at Hospital Pengajar Universiti Putra Malaysia (HPUPM), Hospital Universiti Teknologi Mara (UiTM), and Hospital Serdang. A total of 24 patients were recruited from the Orthopedic Clinics at HPUPM, UiTM Hospital, and Serdang Hospital. They were screened and selected based on the inclusion and exclusion criteria. Patients who met any of the exclusion criteria were excluded from the study. Patient data for pain scores using a 10-cm visual analog scale (VAS) rating system and functional outcomes using the American Orthopaedic Foot and Ankle Society (AOFAS) ankle-hindfoot score system were extracted from the electronic medical records and hospital information systems. All VAS and AOFAS ankle-hindfoot scores were extracted and tabulated pre and post-procedure at one, three, and six months. Demographic information was obtained from the patient's clinical notes, including age, comorbidities, history of previous trauma or surgery, previous ligament injuries, foot and ankle operations, allergy history, pregnancy, and types of previous conservative treatments that the patient underwent. Before undergoing pulsed RFNA, for each patient, conservative treatment was attempted for at least six months and included the use of oral anti-inflammatory medications, local corticosteroid injections, foot orthotics, physical therapy, padding and strapping and the use of a plantar fascia night splint. When conservative treatment failed and the plantar heel pain persisted, the patient was offered pulsed RFNA for the treatment of recalcitrant plantar fasciitis. Pulsed RFNA was attempted on consecutive patients who failed six months or more conservative treatment for their plantar heel pain. All pulsed RFNA procedures were performed by three foot and ankle fellowship-trained surgeons from their respective hospitals. Any discrepancies were resolved by consensus among the surgeons.

The inclusion criteria for this study included patients who experienced heel pain specifically located at the medial calcaneal tubercle of the heel for more than six months, a positive windlass test, and classical symptoms of heel pain such as pain first thing in the morning and after prolonged activity. Additionally, eligible patients had undergone at least two of the following conservative treatment options: stretching exercises and ice treatment, oral anti-inflammatory medication with a heel pad, physical therapy, night splints, steroid injection, arch supports, or taping/strapping. Participants were required to be at least 18 years of age.

Patients were excluded from the study if they had a history of trauma or fracture of the calcaneus, peripheral vascular ischemia, an open wound or infection in the heel area/region, calcaneal lesions including benign tumors, severe fat pad atrophy, calcaneal bursitis, or skin abnormalities around the heel. Pregnant individuals, those under 18 years of age, and those with a local or systemic infection on the date when the procedure was to be performed were also excluded. Additionally, patients with an allergy to local anesthetics or steroids were not eligible for inclusion.

**Figure 1:**
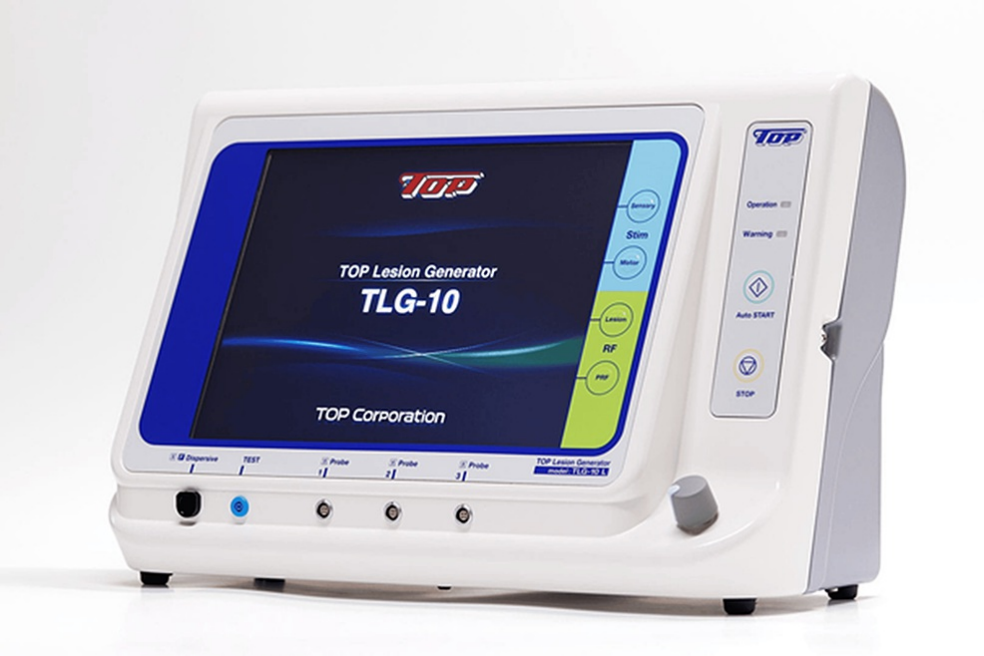
RF Lesion Generator Top TLG-10 STP RF: Radiofrequency; TLG: Top lesion generator; STP: Sluijter Teixeira Pulse

**Figure 2:**
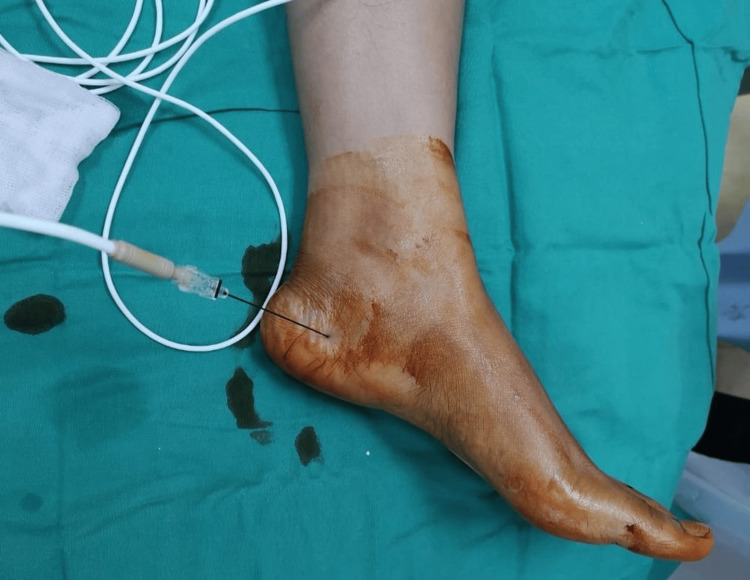
Pulsed RFNA over the left foot The clinical picture shows a pulsed radiofrequency nerve ablation being conducted. A cannula was inserted over the medial aspect of the left heel, specifically at the inferior calcaneal nerve, after using sensory stimulation to identify the nerve prior to the RFNA. RFNA: Radiofrequency nerve ablation

Before entering the procedure room, each patient’s heel was palpated, and the area or areas of maximum tenderness were indicated with a marking pen. Following identification of the specific areas of tenderness, the patient was administered an intradermal injection of local anesthesia in the supine position, and the involved foot was prepped and draped in a sterile fashion. A 22-gauge cannula with a solid stylet was inserted into the first marked site. Sensory stimulation was performed to identify the location of the target nerve, the medial calcaneal nerve, and the inferior calcaneal nerve. At this point, local anesthesia is applied to the target nerve to relieve pain during RFNA. It is through this cannula that the insulated radiofrequency probe is inserted and then connected to the radio energy generator (RF Lesion Generator Top TLG-10 Sluijter Teixeira Pulse (STP), Cosman Medical, Burlington, United States) (Figure 1). Next, pulsed RFNA is applied at a set temperature of 42°C and voltage of 45V and delivered for four minutes to the targeted nerve (Figure 2). Following the injections, small bandages (coverlets) were applied to the involved sites on the heel, and the patient was allowed to return to shoe gear immediately following the procedure and was able to ambulate on the foot as tolerated. Patients were given nonsteroidal anti-inflammatory drugs upon discharge, and the patient was instructed to ambulate as desired and to follow up at scheduled visits. Patients were also instructed to continue with supportive shoe gear and orthoses and to continue physiotherapy as usual.

The statistical analysis was conducted using IBM SPSS Statistics for Windows, Version 26 (Released 2019; IBM Corp., Armonk, New York, United States). Descriptive analysis of demographic, VAS, and AOFAS data was presented as mean (standard deviation) for continuous variables, frequency (n), and percentage (%) for categorical variables. Due to the non-normal distribution of variables, non-parametric tests were employed. Spearman's correlation coefficient and Mann-Whitney tests were used to assess factors associated with VAS and AOFAS ankle-hindfoot scores among patients who underwent pulsed RFNA at different intervals. The efficacy of pulsed RFNA treatment was determined using the Friedman test, with outcomes having a p-value <0.05 considered statistically significant.

This research has obtained research and ethical approval from 1) Medical Research and Ethics Committee (MREC, Ref: 22-02841-C7L dated February 20, 2023) and is registered with the National Medical Research Register (NMRR); 2) Research Ethics Committee UiTM (REC UiTM, Ref: REC/03/2023 (OT/MR/4) dated March 6, 2023); 3) Ethics Committee for Research involving Human Subjects of Universiti Putra Malaysia (JKEUPM, Ref: JKEUPM-2023-215 dated March 31, 2023).

Approval to conduct the study within the facilities of HPUPM, Serdang Hospital, and UiTM Hospital was also obtained from the Director and Head of the clinical research center of each hospital.

## Results

**Figure 3:**
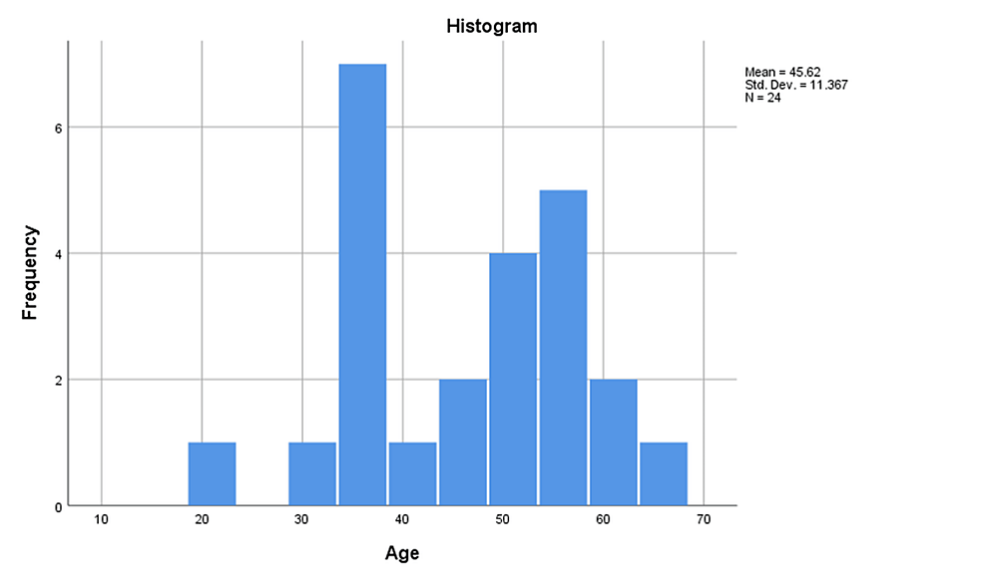
Age distribution chart of the study population (N=24) N: Total number of participants; Std. Dev.: Standard deviation

**Figure 4:**
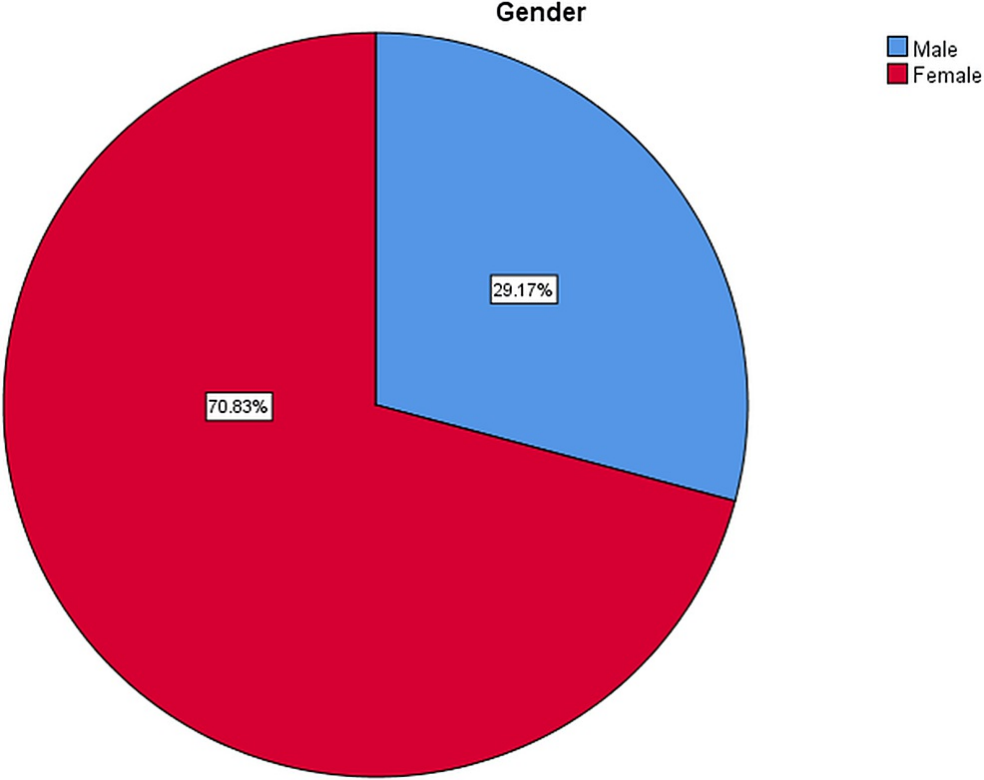
Pie chart showing the percentage distribution of gender Blue: 29.17% of the participants were male; red: 70.83% of the participants were female

**Figure 5:**
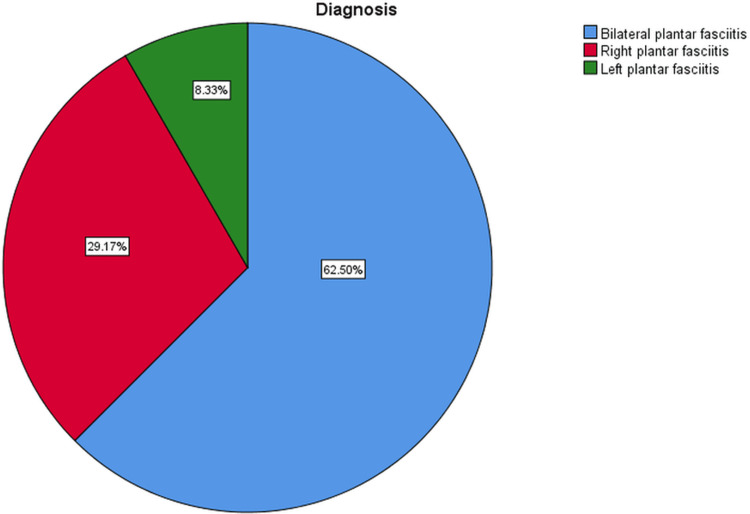
Pie chart showing the percentage distribution of diagnoses Blue: 62.50% had bilateral plantar fasciitis; red: 29.17% had right plantar fasciitis; green: 8.33% had left plantar fasciitis

The presented data in Table 1 pertains to the demographic attributes of the study's participants, with a total sample size of 24 individuals (39 feet). The mean age of the participants was 45.6 years, with a standard deviation (SD) of 11.37 years (Figure 3). The age range of the participants was from 21 years to 64 years. In terms of gender distribution, 29.2% (seven individuals) identified as male, while the majority, constituting 70.8% (17 individuals), identified as female (Figure 4). Meanwhile, regarding the diagnosis of plantar fasciitis, the participants were categorized into different groups based on the affected side. The largest subgroup consisted of individuals with bilateral plantar fasciitis, representing 62.5% (15 individuals) of the sample. Those with right-sided plantar fasciitis accounted for 29.2% (seven individuals), while the smallest subgroup, with left-sided plantar fasciitis, comprised 8.3% (two individuals) of the total sample (Figure 5).

**Table 1:** Demographic characteristics of the study participants (N=24) SD: Standard deviation; n: Frequency; N: Total number of participants

Characteristic	Mean (SD)	n (%)
Age (years)	45.6 (11.37)	
Gender		
Male		7 (29.2)
Female		17 (70.8)
Diagnosis		
Bilateral plantar fasciitis		15 (62.5)
Right plantar fasciitis		7 (29.2)
Left plantar fasciitis		2 (8.3)

Table 2 presents the VAS pain scores and AOFAS ankle-hindfoot functional scores of patients who underwent pulsed RFNA at different time intervals, categorized by right and left foot. The scores are used to gauge the outcomes of the procedure in terms of pain and functionality. Before the pulsed RFNA procedure, patients reported experiencing notable pain, with average VAS scores of 7.8 (0.97) for the right foot and 7.5 (1.55) for the left foot. Their functional status, as indicated by AOFAS ankle-hindfoot scores, averaged 59.0 (9.67) for the right foot and 59.7 (10.37) for the left foot.

**Table 2:** Characteristics of VAS and AOFAS ankle-hindfoot scores among patients who underwent RFNA at different intervals * Value presented in mean (SD). VAS: Visual analog scale; AOFAS: American Orthopaedic Foot and Ankle Society; RFNA: Radiofrequency nerve ablation; SD: Standard deviation

	VAS		AOFAS	
Characteristic*	Right foot	Left foot	Right foot	Left foot
Pre-procedure	7.8 (0.97)	7.5 (1.55)	59.0 (9.67)	59.7 (10.37)
one-month post-procedure	1.8 (0.61)	1.7 (0.59)	88.1 (7.07)	88.0 (7.17)
three-month post-procedure	2.2 (1.51)	2.4 (1.58)	89.5 (8.14)	89.9 (8.26)
six-month post-procedure	1.4 (0.90)	1.5 (0.87)	94.2 (7.32)	94.0 (6.03)

At the one-month mark following the procedure, a substantial reduction in pain was observed, with VAS scores decreasing to 1.8 (0.61) for the right foot and 1.7 (0.59) for the left foot. This improvement in pain was accompanied by enhanced functional outcomes, as denoted by elevated AOFAS ankle-hindfoot scores of 88.1 (7.07) for the right foot and 88.0 (7.17) for the left foot. After three months post-procedure, pain scores remained relatively low, with VAS scores of 2.2 (1.51) for the right foot and 2.4 (1.58) for the left foot. Correspondingly, patients' functional status continued to be favorable, with AOFAS ankle-hindfoot scores of 89.5 (8.14) for the right foot and 89.9 (8.26) for the left foot. At the six-month juncture following the pulsed RFNA procedure, patients reported a further reduction in pain, with VAS scores decreasing to 1.4 (0.90) for the right foot and 1.5 (0.87) for the left foot. Additionally, their functional status demonstrated substantial improvement, with AOFAS ankle-hindfoot scores of 94.2 (7.32) for the right foot and 94.0 (6.03) for the left foot.

Table 3 presents an exploration of demographic factors about the VAS pain scores and AOFAS ankle-hindfoot functional scores among patients who underwent pulsed RFNA at varying time intervals. The age group underwent statistical analysis using Spearman's correlation test, which revealed that age did not achieve statistical significance in all the intervals (p>0.05). These findings suggest that age did not significantly impact the VAS and AOFAS ankle-hindfoot scores across the different intervals. In contrast, the gender and diagnosis groups were assessed using the Mann-Whitney U test, and the results showed that neither gender nor the specific diagnosis of plantar fasciitis exerted a discernible influence on the scores across the different intervals, as the p-values consistently remained above the significance threshold (p>0.05).

**Table 3:** Demographic factors associated with the VAS and AOFAS ankle-hindfoot scores among patients who underwent RFNA at different intervals (a): Spearman’s correlation coefficient test with rs value presented; (b): Man-Whitney U test with mean (SD) value presented. All the values had p-values more than 0.05 (not statistically significant). VAS: Visual analog scale; RF: Right foot; LF: Left foot; PF: Plantar fasciitis; AOFAS: American Orthopaedic Foot and Ankle Society; RFNA: Radiofrequency nerve ablation

	Pre-procedure				One-month post procedure				Three-month post procedure				Six-month post procedure			
	VAS		AOFAS		VAS		AOFAS		VAS		AOFAS		VAS		AOFAS	
	RF	LF	RF	LF	RF	LF	RF	LF	RF	LF	RF	LF	RF	LF	RF	LF
Age (years)^a^	-0.21	-0.14	-0.06	-0.15	0.05	0.08	-0.31	-0.17	-0.48	-0.41	0.10	0.24	-0.32	-0.21	0.07	0.08
Gender																
Male	7.5	7.5	54.0	54.0	2.0	2.0	90.0	90.0	3.0	3.0	87.0	87.0	1.0	1.0	95.0	95.0
Female	8.0	8.0	54.0	54.0	2.0	2.0	88.0	88.0	2.0	2.0	90.0	90.0	1.0	1.0	90.0	90.0
Diagnosis^b^																
Bilateral PF	8.0	8.0	54.0	54.0	2.0	2.0	88.0	88.0	2.0	2.0	88.0	88.0	1.0	1.0	90.0	90.0
Right PF	8.0		54.0		2.0		90.0		1.0		90.0		1.0		100.0	
Left PF		9.0		65.0		1.0		90.0		1.5		95.0		1.0		100.0

**Figure 6:**
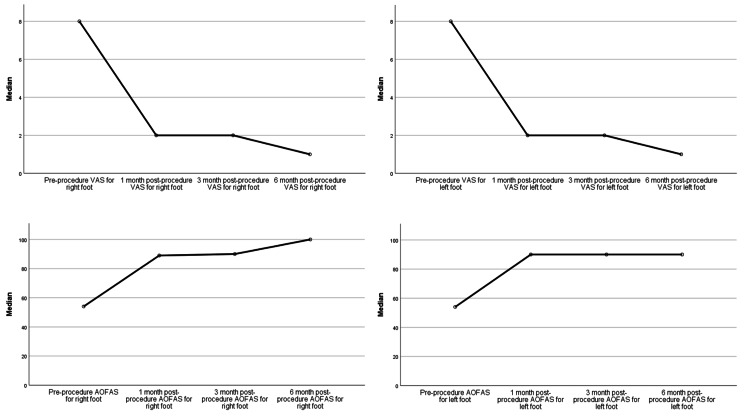
Overall RFNA treatment outcomes among patients with recalcitrant plantar fasciitis VAS: Visual analog scale; AOFAS: American Orthopaedic Foot and Ankle Society; RFNA: Radiofrequency nerve ablation

Table 4 presents a comprehensive evaluation of the effectiveness of pulsed RFNA treatment in mitigating pain and enhancing functional outcomes among patients grappling with recalcitrant plantar fasciitis. To analyze the data in this table, the Friedman test was utilized to account for repeated measures across different time intervals. The overall results indicate a remarkable reduction in pain and a noteworthy enhancement in functional outcomes following the pulsed RFNA treatment (Figure 6). Both the right and left foot VAS pain scores exhibited substantial declines from pre-procedure to subsequent intervals (one-month, three-month, and six-month post-procedure) with p-values of less than 0.001, signifying a highly significant effect. The same pattern of improvement is observed in the AOFAS ankle-hindfoot scores, a metric reflecting functional improvement. The AOFAS ankle-hindfoot scores for both right and left feet showed significant elevations across the post-procedure intervals (one-month, three-month, and six-month) compared to the pre-procedure scores (p<0.001). These findings underscore the significant and consistent positive impact of pulsed RFNA treatment on pain reduction and functional improvement in patients with recalcitrant plantar fasciitis.

**Table 4:** Efficacy of RFNA treatment in reducing pain and improving functional outcomes among patients with recalcitrant plantar fasciitis (a) Values presented as median. *p-value for Friedman test (non-parametric repeated measures test). VAS: Visual analog scale; AOFAS: American Orthopaedic Foot and Ankle Society; RFNA: Radiofrequency nerve ablation

Characteristics^a^	Pre-procedure	One-month post-procedure	Three-month post-procedure	Six-month post-procedure	*p
Overall					
Right foot VAS	8.0	2.0	2.0	1.0	<0.001
Left foot VAS	8.0	2.0	2.0	1.0	<0.001
Right foot AOFAS	54.0	89.0	90.0	100.0	<0.001
Left foot AOFAS	54.0	90.0	90.0	90.0	<0.001
Bilateral plantar fasciitis					
Right foot VAS	8.0	2.0	2.0	1.0	<0.001
Left foot VAS	8.0	2.0	2.0	1.0	<0.001
Right foot AOFAS	54.0	88.0	90.0	90.0	<0.001
Left foot AOFAS	54.0	88.0	90.0	90.0	<0.001
Right plantar fasciitis					
Right foot VAS	8.0	2.0	1.0	1.0	0.001
Right foot AOFAS	54.0	90.0	100.0	100.0	<0.001
Left plantar fasciitis					
Left foot VAS	9.00	1.00	1.50	1.00	0.145
Left foot AOFAS	65.0	90.0	95.0	100.0	0.139

**Figure 7:**
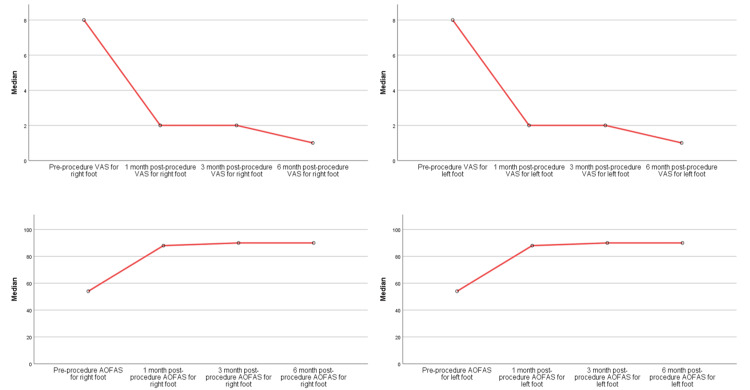
RFNA treatment outcomes among the bilateral recalcitrant plantar fasciitis patients VAS: Visual analog scale; AOFAS: American Orthopaedic Foot and Ankle Society; RFNA: Radiofrequency nerve ablation

**Figure 8:**
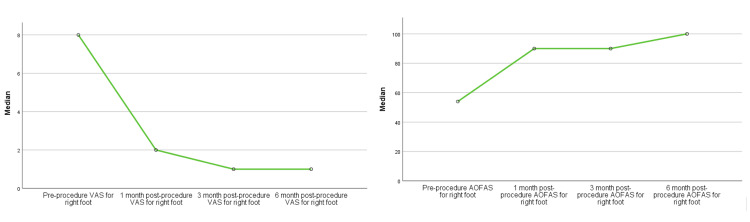
RFNA treatment outcomes among the right recalcitrant plantar fasciitis patients VAS: Visual analog scale; AOFAS: American Orthopaedic Foot and Ankle Society; RFNA: Radiofrequency nerve ablation

**Figure 9:**
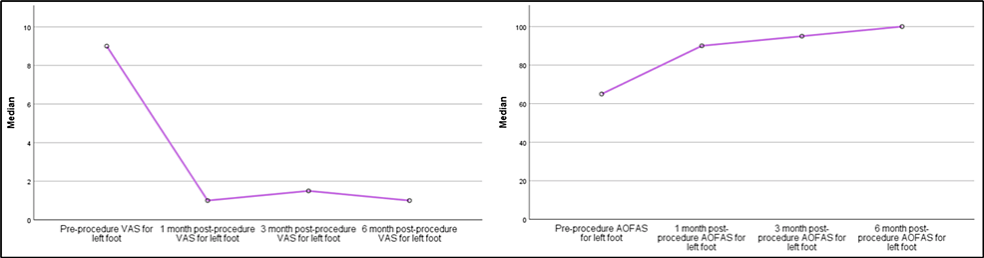
RFNA treatment outcomes among the left recalcitrant plantar fasciitis patients VAS: Visual analog scale; AOFAS: American Orthopaedic Foot and Ankle Society; RFNA: Radiofrequency nerve ablation

Furthermore, the study distinguished between different presentations of plantar fasciitis, encompassing bilateral, right-sided, and left-sided cases. The analysis indicated that the treatment's efficacy in terms of pain reduction and functional enhancement was consistent across these subgroups. Notably, patients with bilateral (Figure 7) and right-sided (Figure 8) plantar fasciitis exhibited remarkable improvements, as indicated by the reduction in VAS scores and the increase in AOFAS ankle-hindfoot scores over time (p<0.001). However, for individuals with left-sided plantar fasciitis (Figure 9), although there were improvements in VAS scores and AOFAS ankle-hindfoot scores, these improvements did not reach statistical significance at all post-procedure intervals (p>0.05) which could be due to the small number of patients with left recalcitrant plantar fasciitis (n=2).

## Discussion

This study offers a comprehensive assessment of the effectiveness of pulsed RFNA in addressing recalcitrant plantar fasciitis, building upon a body of related research in this domain. Previous studies have delved into diverse treatment modalities for plantar fasciitis, shedding light on the potential advantages of pulsed RFNA.

In a prospective comparative study led by Osman and colleagues in 2016, the efficacy of pulsed and TRF on the medial calcaneal nerve for chronic refractory plantar fasciitis was investigated. Their findings emphasized pulsed RFNA as a secure and effective method for alleviating pain associated with chronic plantar fasciitis, particularly due to its quicker onset of effective analgesia compared to TRF [1].

In another study in 2013, Erken and collaborators conducted a prospective investigation into percutaneous RFNA for chronic plantar fasciitis in 29 patients. The study demonstrated that RFNA, specifically targeting the calcaneal branches of the inferior calcaneal nerve, is an effective treatment for chronic heel pain when conservative options fail [2].

A retrospective analysis in 2009 by Liden and colleagues focused on 22 patients undergoing percutaneous RFNA for prolonged, moderate to severe heel pain associated with plantar fasciitis. Their results strongly endorsed RFNA as an excellent modality, especially when conservative measures proved insufficient, suggesting it as an alternative to more invasive open surgical procedures for plantar fasciitis treatment [3].

In 1997, Ronald and colleagues examined the effectiveness of radiofrequency lesioning in treating plantar fasciitis in 39 patients. The findings indicated that radiofrequency lesioning is a highly effective technique for relieving plantar fasciitis, proposing it as a minimally invasive alternative for patient’s refractory to conservative measures before considering surgical intervention [4].

A 2020 study led by Yuan and colleagues compared open plantar fascia release and percutaneous RFNA for intractable plantar fasciitis in 31 patients. The conclusion favored percutaneous RFNA, primarily due to its shorter operative and postoperative recovery times [5].

In 2019, Turhan and Arican performed a comparison of three treatment modalities for chronic plantar fasciitis in 48 patients, including corticosteroid injection, extracorporeal shock wave therapy, and RFNA. Their results showed no significant differences in outcomes among these treatments, indicating substantial improvements in managing chronic plantar fasciitis with all three modalities [6].

A 2016 study by Arslan and colleagues explored the treatment of chronic plantar heel pain with RFNA of the first branch of the lateral plantar nerve and medial calcaneal nerve branches in 37 patients. The study suggested that, with precise diagnosis and appropriate technique application to painful points, chronic plantar heel pain can be successfully treated with RFNA [7]. Thapa and Ahuja described two case reports where PRF to the medial calcaneal nerve was effective in treating refractory plantar fasciitis pain [8].

Approximately 11% to 15% of cases with chronic plantar fasciitis require treatment [9]. The standard treatment is conservative, yielding successful results in 90% to 95% of the cases and proving to be sufficient in many cases. However, minimally invasive treatment modalities, such as pulsed RFNA, have some potential advantages, including being well-tolerated and having fewer side effects. RFNA works by dissipating heat from an active electrode [9]. It is used in the treatment of numerous clinical conditions, including trigeminal neuralgia, lumbar disc herniation, coronary vascular disease, cardiac arrhythmia, cervical pain syndrome, essential tremor, neuroma, verrucae, and ingrown toenails [9].

Several previous studies have indicated that conservative management, including biomechanical control or anti-inflammatory drug therapy, often proves effective in addressing heel pain. When these approaches fail to yield positive outcomes, more invasive interventions such as surgery are commonly considered. Although controlled studies with placebos are not available, surgical procedures involving partial or complete release of fascial bands and removal of the calcaneal spur have shown a high success rate, reaching over 70% success in some studies [10]. However, it's important to note that partial or complete plantar fascial release comes with its set of challenges. Historically, a small percentage of these surgical releases have been associated with issues like cuboid compression syndrome, iatrogenic pes planus, and calcaneal nerve injuries [10]. Moreover, complications at the surgical site, such as hematoma, infection, wound separation, and postoperative calcaneal fractures, have been observed.

For the current study, the hypothesis was centered around the application of pulsed RFNA for the treatment of recalcitrant plantar heel pain associated with plantar fasciitis. The results of this study, which included 24 participants (39 feet), revealed a substantial improvement in both pain reduction and functional outcomes following the pulsed RFNA procedure. Before undergoing pulsed RFNA, patients reported experiencing substantial pain, with average VAS scores of 7.8 for the right foot and 7.5 for the left foot. Their functional status, as measured by AOFAS ankle-hindfoot scores, was also quite low. However, at the one-month, three-month, and six-month post-procedure intervals, there was a remarkable reduction in pain and a significant increase in functional scores. These improvements were statistically significant, with p-values of less than 0.001, underscoring the effectiveness of pulsed RFNA in alleviating pain and improving functionality for patients with recalcitrant plantar fasciitis.

In addition, the study explored the potential impact of demographic factors, such as age, gender, and the specific diagnosis of plantar fasciitis, on the treatment outcomes. Notably, age, gender, and the specific diagnosis did not demonstrate statistically significant effects on the VAS and AOFAS ankle-hindfoot scores across different intervals, highlighting the consistent benefits of pulsed RFNA treatment across these demographic factors.

Furthermore, the study distinguished between different presentations of plantar fasciitis, categorizing them as bilateral, right-sided, or left-sided cases. The outcomes revealed that patients with bilateral and right-sided plantar fasciitis experienced significant improvements in both pain reduction and functionality. However, those with left-sided plantar fasciitis exhibited improvements that, though observable, did not attain statistical significance, likely due to the limited sample size within this subgroup. There were no complications encountered during the study. However, two participants experienced persistent symptoms with mild pain reduction and suboptimal functional outcomes, likely due to the severity of the disease and inadequate response to the treatment provided.

Limitations

The present study had several limitations. These included its retrospective design, a relatively small sample size, and the absence of a long-term assessment of the procedure's effectiveness. Additionally, the study did not explore the impact of various factors, such as activity level and daily standing duration, which could potentially influence the outcomes. These aspects warrant consideration in future research endeavors. Furthermore, the lack of a control or comparison group and potential bias, such as selection bias, are also limiting factors that should be acknowledged.

## Conclusions

In summary, the data presented in this study provide valuable insights into the outcomes of pulsed RFNA treatment for patients with recalcitrant plantar fasciitis. The study aligns with and extends the findings of previous research on pulsed RFNA's effectiveness in treating this condition. The results emphasize that pulsed RFNA can provide substantial pain relief and functional improvement for patients who have not responded to conservative treatments. This knowledge can be valuable for healthcare professionals and patients seeking alternative solutions for this challenging condition. However, future research with larger sample sizes is warranted to further confirm and expand upon these findings. These insights reinforce the evolving role of pulsed RFNA as a minimally invasive procedure in the treatment of recalcitrant plantar fasciitis, offering a potentially promising option to alleviate the suffering associated with this condition, for which no standard treatment guidelines exist.
